# Extensive Abdominal Wound, Etc.

**Published:** 1875-12

**Authors:** Henry R. Rogers

**Affiliations:** Dunkirk, N. Y.


### EXTENSIVE ABDOMINAL WOUND,

### WITH PROTRUSION OF THE BOWELS AND LACERATION AND REMOVAL OF A PORTION OF THE LIVER.

By HENRY R. ROGERS, M.D., Dunkirk, N. Y.

The interesting case of extensive abdominal wound, reported recently by Dr. Carmichael, revives the memories of a similar one in my professional charge, several years since.

The subject of the accident was Mr. Samuel Erbes, a carpenter by trade, aged about 36 years. Whilst standing in front of a circular saw in rapid motion, he was struck by a missile thrown by the saw, and severely wounded in the abdominal wall.

On my arrival a few minutes subsequent to the injury, I found the man unconscious, pallid, pulseless, and with respiration almost extinct. The wound of the abdominal wall commenced at a point two inches above the umbilicus and passed to the left nearly horizontally to the extent of eight inches. Projecting through the wound was a large mass of intestines and a portion of the liver, attached by a small pedicle of its tissue. This latter, when detached, weighed nearly two ounces, and in form was quite irregular. The intestines after being cleaned were returned to the cavity of the abdomen, the wound was secured by sutures and strips of adhesive plaster, and the parts sustained by a comfortably fitting bandage. The dressings being protected from moisture by a large piece of oiled silk, *the wound*

*was kept as cool as possible for several days by the application of cloths, wrung out from cold water*, applied as occasion required. *Perfect exemption from pain was secured by the exhibition of opiates*, subcutaneously at first, later by the mouth. This treatment was continued four days without other change than an enema for moving the bowels.

During this period no untoward symptoms were present, no fever, no pain, and scarcely any discomfort other than tenderness over the abdomen on motion and on pressure. The wound was then examined and found to be healed throughout its whole extent by first intention. The sutures were removed, the strips and bandage re-applied, and no further professional attention was required on account of the wound.

The sixth day the patient, contrary to my urgent and emphatic counsel, walked about his apartments. This imprudence was followed by no serious results, and recovery was soon complete.

These cases are not without practical value to the profession in sustaining the position now being assumed in regard to the treatment of inflammation of whatever kind and wherever occurring.

The old system of treatment by alteratives, is a thing of the past. *Requiescat in pace.*

The treatment of acute inflammation by opiates has its foundation in reason and philosophy. The tell-tale nerves in irritated or inflamed parts carry information of their condition quickly to the brain and the response is quickly returned. Currents of blood are hastened to the scene of disturbance, yet more seriously disquieting the nerves of the parts implicated, and if this process remains unchecked, a seated inflammation is the natural result. If, however, instead of permitting the indications of lesion to be carried to the brain, we quiet the sensibilities of the offended nerves by such agents as may be suitable, the process of inflammation will be checked or arrested. This fact in practical medicine is well known to the intelligent and reading members of the profession, yet it is too little understood and appreciated by the vast majority of its members.

